# Surveillance of human papillomavirus through salivary diagnostics - A roadmap to early detection of oropharyngeal cancer men

**DOI:** 10.1016/j.tvr.2024.200278

**Published:** 2024-03-03

**Authors:** Akila Wijesekera, Chameera Ekanayake Weeramange, Sarju Vasani, Liz Kenny, Emma Knowland, Jayampath Seneviratne, Chamindie Punyadeera

**Affiliations:** a Griffith Institute for Drug Discovery, School of Environment and Science, Griffith University, Queensland, Australia; b Royal Brisbane and Women's Hospital, Queensland, Australia; c Faculty of Medicine, University of Queensland, Queensland, Australia; d Metro North Sexual Health and HIV Service, Queensland, Australia; e School of Dentistry, University of Queensland, Queensland, Australia; f Menzies Health Institute Queensland, Griffith University, Gold Coast, Australia

**Keywords:** Oropharyngeal cancer, Screening, OPC, HPV, Human papillomavirus, Persistence

## Abstract

Human papillomavirus (HPV) is the most common sexually transmitted disease. Certain strains have the potential to cause malignancy in multiple anatomical sites if not cleared by the immune system. In most infected people, HPV is cleared within two years. However, HPV may persist in susceptible individuals with certain risk factors, eventually leading to malignancy. New evidence suggests that over 75% of all oropharyngeal cancers (OPC) are directly attributable to HPV. It is estimated that prophylactic HPV vaccination alone may take at least 25 years to have a significant impact on reducing the incidence of OPC. The temporal link between detection of oral HPV, persistence of the infection and the subsequent development of OPC have been well established. Moreover, men have threefold higher risk than women for acquiring HPV-OPC. This comprehensive review focuses on OPC development in men, highlighting the risk factors associated with malignant transformation of HPV-OPC. Current evidence is insufficient to determine whether early identification of at-risk demographics, screening, and prompt diagnosis result in improved outcomes. Hitherto, the effectiveness of an oral HPV screening program in this regard has not been investigated. Nevertheless, the potential to emulate the success of the cervical screening program remains a very real possibility.

## 1 Introduction

### 1.1 Discovery of HPV as a risk factor for the development of cancer

Harald Zur Hausen was the first to challenge in the 1970s the widely accepted concept that cervical cancer is caused by herpes simplex virus-2 (HSV-2). On the contrary, Zur Hausen proposed a theory that cervical cancer is caused by human papillomavirus (HPV), not HSV-2. The first microscopic observation of HPV was reported by Strauss et al. in 1949, where it was isolated in skin papillomas [1]. More detailed observations on HPV DNA were reported by Crawford and Crawford in 1963 [2]. However, it was only Harald Zur Hausen's pioneering work that placed HPV into the context of cancer-causing virus, for which he received the Noble Prize in Physiology or Medicine in 2008.

Today, we know that HPV is a small double-stranded DNA virus and over 200 different sub-types of HPV have been identified [3]. The vast majority of them do not cause symptomatic disease. Harald Zur Hausen hypothesized that certain strains of HPV, such as HPV-16 and HPV-18 are capable of causing cancer (i.e. oncogenesis) based on his work on genital warts and cervical cancer. Subsequent studies demonstrated the possibility of differentiating between ‘benign, warty’ causing strains of HPV that do not progress to cervical cancer and oncogenic strains that do advance into cervical cancer. Hence, it was evident that only a specific subset of high-risk HPV (hrHPV) is associated with the development of cancers [4]. hrHPV is not only implicated in the development of cervical cancer, but also in other areas of the body such as anal, penile, and oropharyngeal cancers. In women, almost all cervical cancers are associated with hrHPV, with the virus being detected in 99.7% of cases [5]. In men, it is typically implicated in penile and anal cancers. In more recent years, the role of hrHPV in oropharyngeal cancers (OPC) has been increasingly recognized [6]. Alarmingly, hrHPV strains are now implicated in approximately 75% of OPCs. With a stark discrepancy between men and women diagnosed with HPV-OPC, some reports documented as high as 6-fold and more frequently at least 3-fold respectively [[7], [8], [9], [10]]. Men diagnosed with HPV-OPC, are younger and tend to be non-smokers compared to non-HPV OPC [8,9,11]. This has raised concerns among clinicians globally, as the incidence of OPCs is expected to double in the next decade [12].

In Australia, the quadrivalent HPV vaccine [6,11,16,18] has been a part of the routine vaccination schedule for girls since 2007 and then for boys since 2013, the uptake of which is among the highest in the world. However, men over the age of 25 are unlikely to have received this preventative HPV vaccine and as a consequence, the vaccination program is unlikely to have an impact on OPC incidence currently [13]. There is undisputed agreement that the HPV vaccination stands as a significant breakthrough in preventive medicine. Numerous studies have demonstrated its effectiveness in diminishing HPV-related cancers, particularly cervical cancer, and in turn, it has played a crucial role in saving countless lives. But there is new literature emerging that in the oropharynx, perhaps the vaccination isn't as efficacious against hrHPV. This was highlighted recently by De Souza et al. 2023, who conducted a study on men and women participants aged between 18 and 70 years old in Australia, with a total of 911 salivary samples being analysed. They found that of those participants who carried oral HPV, 53% of them carried hrHPV, but most interestingly, they found no difference in this incidence between the vaccinated and unvaccinated groups [14].

It is likely that vaccination is effective in the oropharynx to some extent, the true efficacy is yet to be seen, but it has been demonstrated in controlled period analyses that the impact of the vaccination in halting the increasing incidence of OPC will take over 25 years [15]. This window of time is where the advent of a screening process may be necessary.

### 1.2 Transmission of HPV

The primary route of HPV transmission is skin-to-skin or skin-to-mucosa contact. Thus, the majority of HPV infections are acquired through sexual contact. In fact, HPV is the most common sexually transmissible infection (STI) in the world, with 80% of sexually active individuals expected to be infected at least once by age 45 [16]. Although HPV infections aren't actively monitored, globally, from 1990 to 2019 the combined incidence of other monitored STIs: syphilis, chlamydia, gonorrhoea, trichomoniasis and genital herpes increased by 58.15% from 486.77 million to 769.85 million, with men having an overall high incidence [17]. The World Health Organization estimates that 300 million women will have a genital HPV infection in 2022 [18].

HPV is primarily spread through vaginal intercourse, however, other sexual activities such as oral and anal sex could also transmit the virus to one another. Anal sex has been identified as the most strongly associated sexual behaviour for contracting HPV [19,20]. Further, the risk of contracting HPV is increased with the number of sexual partners [21]. A prospective trial involving 87 virgin men in Brazil, Mexico and the United States found that 45.5% of the people who engaged in active sexual behaviour acquired genital HPV within 24 months [21]. It is noteworthy that the host immune system is able to clear the virus in some cases whereas in other cases HPV could persist for an extensive period of time. The aforementioned study found that of the people who engaged in penetrative sex in the study period, 27.8% had persistent genital HPV infection, compared to those who abstained from penetrative sex, where only 5.9% of subjects had persistent infections. This study confirmed the hypothesis that increased sexual activity places people at increased risk of persistent genital HPV infection.

### 1.3 Pathogenesis of HPV driven malignancy

Cutaneous warts and mucosal papilloma are caused by low-risk HPV (lrHPV) strains such as 2, 6, and 11. The likelihood of malignant transformation of these lesions is rare [22]. In contrast, there are over 20 high-risk HPV (HrHPV) strains with known carcinogenic potential. Particularly, strains 16 and 18 are implicated in over 70% of all cervical cancers [23].

HPV infects the epithelial lining via micro-wounds that expose the basal layer, which contains the actively replicating population of epithelial cells. Following entry, the virus maintains a low number of extrachromosomal copies, approximately 20–100, within the infected basal cells. This initial viral genome amplification is enabled by HPV E1 and E2 proteins. When the basal cells divide and commence differentiation, the virus enters the proliferative phase of its life cycle, during which exponential amplification of viral genomes occurs under the influence of HPV E5, E6 and E7 [24]. HPV-driven cellular transmission can be mainly attributable to these proteins and hence they are also known as HPV oncoproteins. E7 binds to the retinoblastoma class of tumour suppressor proteins, which serves to limit the action of the E2F transcription factor [25]. E6 directly binds to the p53 tumour suppressor protein, deactivating it, leading to high turnover of p53. Although considered non-essential for malignant transformation, E5 also plays a supportive role by interrupting host defences and promoting cell proliferation [24,26,27]. In the setting of p53 being deactivated and removal of the suppressive action of pRb, there is an environment that is perfectly suited to uninhibited cellular proliferation [28]. This prolongation of the replication period ultimately leads to an accumulation of mutations which in turn, sporadically give rise to dysplastic transformation [29,30]. The pRb and p53 pathways are not the only mechanism by which E6 and E7 exert their oncogenic potential.

E7 can destabilize “pocket proteins” p107 and p130, which regulate, the action of the E2F transcription factors [31]. This leads to cellular transformation by disrupting the normal function of E2F [31]. A by-product of this is the overexpression of p16, a hallmark of HPV-OPC. Additionally, E7 can overcome the inhibitory actions of p21^CIP1^ and p27^KIP1^ which regulate cell cycle arrest in keratinocyte replication, disruption leads to immortalization of the cell and in combination with CDK2 promotion into the S phase of the cell cycle [32].

A lesser-described action of E6 is its role in telomerase reverse transcription (TERT) modulation and activation via a cascade with c-MYC (family of proto-oncogenes). Experimentally, it has been demonstrated that activation of TERT is essential for cellular immortalization and malignant transformation. Such, that TERT expression is detected in up to 90% of all cancers [33]. Additionally, E6 can prevent the host immune system from mounting an adequate response against infected cells via interruption of the interferon response [34].

Although lrHPV types also produce E6 and E7 they are not known to promote genomic instability and cellular transformation as oncoproteins produced by hrHPV [35]. The E6 protein in lrHPV does not bind to the p53 at detectable levels, and E7 binds to the pRb with decreased affinity to that of hrHPV [36]. This distinct difference is what leads to hrHPV having a far higher malignant potential than lrHPV.

HPV16 holds a unique place with regard to its oncogenic potential. It is widely implicated with HPV-OPC as well as cervical cancer. The current evidence suggests that HPV16 is implicated in at least 85% of all HPV-OPC in the oropharynx [37]. Some studies have reported the prevalence of HPV in OPC to be as high as 97% [38]. In contrast, HPV16 is implicated in 52% of cervical cancers, followed by HPV18 in 18% of cases [39]. It is known that specific sublineages of HPV16 have a higher propensity to cause specific variants of cervical cancer. For example, HPV16 D2 has a 28-fold greater chance of causing invasive cervical cancer, whereas HPV16C is strongly associated with CIN3 [40,41].

The sequencing of oropharyngeal tumours to detect HPV16 sublineage differences is still in its early days. Recently Kuhs et al. (2022) demonstrated that site-specific single nucleotide polymorphisms (SNPs) in HPV16 were significantly correlated to prognosis. With median the survival time for patients who had greater than or equal to one SNP being 3.96 years compared to 18.67 years for those without any SNPs [40]. Additionally, it has been found that there are over 9 times more nonsynonymous E6 gene mutations in HPV-OPC compared to cervical cancer [39]. Whether this indicates the E6 oncogenic pathway is more crucial in OPC as opposed to cervical cancer remains unclear. Perhaps, HPV16 possess a unique mechanism for the oncogenic transformation of epithelia, particularly that of oropharyngeal mucosa compared to other HPV strains and anatomical sites. This will be an interesting area to research and warrants further investigation.

### 1.4 Persistence HPV infection

HPV infection alone is not sufficient to cause malignancy rather a more indolent and chronic process is required [42]. A majority of individuals infected with HPV are asymptomatic and the virus may spontaneously clear within 12–24 months [[43], [44], [45]]. If the virus persists without being cleared by the immune system, it may lead to a metaplastic change in the host cell. For example, immunocompromised individuals with previously latent HPV may suddenly develop numerous cutaneous and respiratory lesions [46].

HPV can effectively evade the vast majority of immune responses by simply staying within the superficial layers of the squamous epithelium [37]. HPV can remain inactive inside basal cells in the form of DNA and avoid triggering a significant immune response from the host. This mechanism of evasion is mainly orchestrated by the E2 gene, which actively represses viral antigen expression [47].

The clearance of HPV lesions within the suprabasal compartment is by cell-mediated infiltration of CD8 and CD4 lymphocytes [48]. Langerhans cells (LC) are the most abundant immune modularity cells found in squamous epithelia. HPV disrupts the immune cascade of the LCs at several stages. Firstly, HPV-induced changes to cytokine expression by the suppression of maturation, migration, and cytokine release of LCs. Secondly, HPV reduces the E-cadherin levels required for the interaction between LCs and keratinocytes [49,50]. Thirdly, HPV downregulates the T-cell response of MHC1-based antigen presentation [51]. Lastly, HPV also downregulates the activity of natural killer cells [52]. As a consequence of the immune dysregulations, both hrHPV and lrHPV can effectively evade the host immune response causing a persistent infection.

In order to meet the nutrient and oxygen demand of the proliferating virus in the persistent infection, HPV activates the hypoxia-inducible factor 1 alpha (HIF-1a) [53]. HPV virus E7 protein is able to displace histone deacetylases from HIF-1a increasing angiogenesis and contributing to the growth of the tumour [53]. Combined with the viral-induced masking from the host immune system, the dysplastic cells continue to replicate. However, this alone isn't sufficient for malignant transformation. There needs to be a secondary insult such as activation of K-ras to the cellular genome for metaplasia changes [54].

### 1.5 Surveillance of persistent HPV infections

As HPV-driven cellular transformation is almost always preceded by HPV persistence, continuous monitoring of HPV infections allows the identification of at-risk individuals for developing HPV-associated cancers. Most of the previous studies have only focused on the detection of a limited number of hrHPV strains. Of them, the majority have examined the HPV16 E6 oncoprotein in serum. The data from the PLCO (Prostate, Lung, Colorectal and Ovarian) Cancer Screening Trial USA 1993 to 2001 with over 154,000 participants showed that in 231 patients who went on to develop HPV-OPC (42%), patients were HPV16-E6 seropositive up to 13 years prior to diagnosis of an HPV16 positive tumour [55]. Similarly, the EPIC study (European Prospective Investigation into Cancer and Nutrition Study) 1992 to 2000 showed that HPV16 E6 was detectable 10 years prior to diagnosis of HPV-OPC [46]. They also reported that HPV16 E6 was seropositive 7–8 years prior to diagnosis of non-cervical cancers (anal, penile, vaginal, and vulvar) [56,57].

Most recently, Busch et al. 2022 detected three early-stage HPV-positive OPCs as part of the Hamburg City Health Study, focused on a population-based prospective cohort aged 45–74 years. They reported that serum HPV16 E6 was detectable 3–4 years prior to diagnosis [58]. All these studies serve to show, that surrogate markers for hrHPV infection are readily detectable in blood years prior to the diagnosis of malignancy. Serum oncoprotein testing and antibody testing are certainly promising, it is however an invasive procedure. Patients are more compliant and willing to partake in non-invasive investigations [59].

### 1.6 Oropharynx: a potential reservoir of HPV?

The clinical potential of saliva as a liquid biopsy-based diagnostic and a prognostic tool has extensively been demonstrated clinically. Collection of saliva is a non-invasive, simple, economical, and widely acceptable procedure for many patients. Studies have examined the validity of saliva for detecting HPV, particularly in cohorts who are at risk of developing HPV-related infections and cancers.

Agalliu et al. 2016 showed HPV-16 was detectable in the oral cavity an average of 3.9 years prior to OPC diagnosis using the PLCO data set as well as the American Cancer Prevention Study II nutrition cohort. The study was a nested case-control among 96,650 participants that associated a 22.4-fold increase in the incidence of OPC when adjusted for smoking history and alcohol consumption [60]. Recently, Kang et al. 2020 showed that HPV16 was detectable in the salivary rinses 2.5 years prior to diagnosis of malignancy [61].

The PROGRESS (Prevalence of Oral HPV Infection, a Global Assessment) trial examined 3196 healthy adults, aged between 18 and 60 years, who visited dental clinics. The study revealed an oral HPV prevalence of 6.6% with a 2% prevalence for hrHPV [62]. This translated to 9.1% of men and 4.6% of women having *any oral HPV* (aHPV) infections. More specifically, 3.3% and 1% of the cohort had oral hrHPV. Importantly, it was observed that the prevalence of aHPV and hrHPV was higher among men across all age groups. The highest prevalence (16.8% any HPV and 6.8% hrHPV) was noted in the 51–60-year-old group. There are other studies that show a higher prevalence of oral aHPV in men (12.3%) as opposed to women (9.5%) [63]. In contrast, a US study published in 2022 reported an oral prevalence of 9% in men and 10.5% in women [64]. However, this study focussed predominantly on university-aged students, with 61% of the 394 participants being under the age of 22. Further assessment of data in the cohort aged >23 years old revealed that the prevalence of oral aHPV was 5.6% in females and 8.0% in males.

NHANES data set has been used to evaluate the prevalence of oral HPV. Brouwer et al. 2019 reported an 11% prevalence in men and 4% in women using the 2003 to 2014 NHANES dataset [65]. Sonawane et al. 2017 found analysing the NHANES 2011 to 2014 data in the USA in a non-institutionalized population that the overall oral HPV prevalence in men aged 18–69 was 11.5%, with 7.3% being hrHPV and 3.2% for women with 1.4% hrHPV [66]. A study examining Men who have Sex with Men (MSM) reported a 23.7% prevalence of oral aHPV [67]. Interestingly, another recent study found the prevalence of oral hrHPV to be 12% in MSM and 6.2% in heterosexual men [68].

Our team conducted a trial at the University of Queensland dental school where we recruited 223 participants with poor oral hygiene and periodontal disease [69]. We detected oral HPV-16 DNA in 10 out of 223 participants (4.5%) using NB2 endpoint PCR and Sanger sequencing. Within the HPV-16 DNA-positive individuals, 7 (70%) and 3 (30%) were associated with poor oral hygiene and periodontal disease, respectively. In a second study also carried out at the University of Queensland dental school, oral HPV16 DNA was detected in 12 out of 650 cancer-free individuals (1.8%; 95% confidence interval [CI]: 1.0–3.2) [70]. With 1.6% of women and 2.1% of men returning positive for oral HPV16 (see Table 1).

**Table 1:** Oral HPV prevalence in men.

Study ID	Total Cohort size	Cohort type	Sampling Type	Age (years)	Prevalence of any HPV in Women	Prevalence of any HPV in Men	Prevalence of High-Risk HPV in Men
Bui et al. 2013 [71]	3439	NHANES 2009–2010	Oral rinse	30–69	3.3%	11.70%	–
Kreimer et al. 2013 [72]	1626	Non-institutionalized Public	Oral rinse & gargle	18–73	–	4.40%	1.70%
Sun et al., 2017 [69]	223	Australian dental school	Oral rinse	18–90	5.8%^b^	–	3.6%^b^
Sonawane et al. 2017 [66]	–	NHANES 2011–2014	Oral rinse	18–69	3.2%	11.50%	7.30%
Antonsson et al. 2020 [63]	284	Australian public	Saliva	20–70	9.5%	12.3%	–
Tang et al. 2020 [70]	650	Australian dental school	Oral rinse	18–89	1.6%^b^	–	2.1%^b^
Brouwer et al. 2022 [65]	394	USA College students^a^	Saliva	18 - 50+	10.5%	9.00%	4.8%
Sonawane et al. 2023 [68]	3232	NHANES 2013–2016	Oral rinse	18–59	–	–	6.20%
Giuliano et al. 2023 [62]	3196	USA dental clinic patients	Oral gargle	18–60	4.6%	9.1%	3.3%

a Also included surrounding suburbs general population (no upper age limit was provided), the vast majority of participants <22 years old (61%) and 67% of cohort female.

b HPV16.

## 2 Risk factors for oral HPV infections

### 2.1 Gender

This disparity of oral HPV prevalence rates based on gender is likely secondary to a multitude of factors. Firstly, men have reported to have a higher number of sexual partners compared to women [73]. For example, a study reported average number of sexual partners for men was 11.6 in contrast to 4.6 for women [73]. However, the difference was smaller in the NCHS CDC USA study showing 6.3 partners for men and 4.3 for women [74]. Secondly, men are more likely to acquire HPV from women as female genitalia carry a higher HPV burden as opposed to male genitalia [75]. As an example, a study found that when women have higher HPV-16 viral loads, their male partner had an increased risk of being HPV-16 positive on genital swabs in a cohort of 238 heterosexual couples attending a gynaecology clinic in the Netherlands Moreover, clearance of men's penile lesions was slower if their partner had the same HPV type in partner's cervix [76]. Another study showed, in heterosexual couples, the rate of HPV transmission from the cervix to the penis was >3-fold higher at 17.4/100 person-months compared to the penis to cervix at 4.9/100 person-months [75]. Thirdly, there is emerging evidence that women may have a more robust immune response against HPV than men [77]. According to a comprehensive review report by Klein et al. in 2016, it was observed that women tend to exhibit a more robust immune response and greater activity against hepatitis B and HIV [77].

In a cohort study conducted by Windon et al. in 2019, involving 374 ENT (Ear, Nose, and Throat) outpatient clinic participants in the US, it was demonstrated that women exhibited significantly higher levels of HPV16 and HPV18 L1 antibodies compared to men (odds ratio (OR) = 2.96, 95% confidence interval (CI) = 1.21–7.21 for HPV16, and OR = 2.84, 95% CI = 1.06–7.60 for HPV18). This difference was still present even when controlled for lifetime and recent sexual behaviour [78]. A potential avenue for this may be due to women being able to seroconvert with a higher success rate when exposed to hrHPV through vaginal contact. It is known that there is an abundance of antigen-presenting cells in the female reproductive tract and that it is an inductive site of the mucosal immune system [79]. Whether this translates to a clinically relevant difference in immune response is unclear, but it may be an explanation for the different rates of hrHPV infection seen between men and women.

### 2.2 Sexual behaviour

As previously mentioned, HPV is the most commonly transmitted STI in the world. There is a multitude of studies showing it is transmitted from penetrative sex, whether this be vaginal or anal intercourse. Until recently, the modality for oropharynx infection wasn't considered particularly relevant or studied. It has now been shown that there is direct inoculation of HPV from oral sex into the oropharynx and from mouth to mouth via deep kissing [80]. The odds of developing an oral HPV infection increase with the number of recent oral sex partners and open-mouthed kissing partners [80]. In a case-controlled cohort of university-aged males in the USA, it was revealed that oral HPV infection is more strongly associated with the number of recent oral sex and open-mouthed kissing partners than with recent vaginal sex partners [69]. Similarly, D'Souza et al. 2017 using the NHANES 2009 to 2016 data found that the prevalence of oral hrHPV increased significantly with the number of oral sex lifetime partners from 1.2% (for 0) to 11.1% (for greater than 10). Relevant to that women may have better immunity against hrHPV; the prevalence of oral hrHPV in men was 2.4% (for 0 oral sex partners) to 14.4% (>10 oral sex partners) compared to 0.2% (for 0) to 3% (>10) for women. Furthermore, in men who have ever performed oral sex on another man, the prevalence was 10.2% compared to 5.8% [81]. Furthermore, Giuliani et al. 2020 found that condomless oral sex was the strongest predictor for oral aHPV infection in a cohort of 244 men who have sex with men (42.2% HIV positive) [82].

These previous studies investigated other modalities of sexual transmission after the foundational work by Giuliano et al., 2011; the HIM study, which found that there is a significant increase in genital hrHPV infection with those having a high number of lifetime female sexual partners, up to 50 partners compared to only one sexual partner (hazard ratio (HR) 2.40, confidence interval 1.38–4.18). They also found a significant increase in genital hrHPV in men who engaged in male anal sex for at least 3 partners compared to no male partners (hazard ratio 2.57, confidence interval 1.46–4.49). Further to this, they noted that the clearance of genital hrHPV infection decreased in men with a high number of lifetime female partners [43].

### 2.3 Oral hygiene status

The means by which poor oral hygiene leads to a higher risk of HPV infection is down to an anatomical quirk of the oral cavity. As previously mentioned, HPV infections occur via microtears in the mucosal epithelium which allows penetration of the virus to the basal lamina. This is how infection occurs in the genitalia and similarly in the pharynx. In contrast, in the oral cavity, the periodontal pocket has regions with exposed basal lamina [83]. Thus, the virus is readily able to infect as the presence of microtrauma isn't a prerequisite. Furthermore, chronic inflammatory changes in the region; as seen in periodontitis, further serve to facilitate HPV infection and persistence [84].

Some studies have shown poor oral hygiene is associated with increased risk for oral HPV infection [85]. Some of the indicators of oral health indices such as plaque index, gingival bleeding index and the number of teeth extracted significantly correlated with the presence of hrHPV [85]. National Health and Nutrition Examination Survey conducted by the CDC using a cohort of 3439 participants found oral aHPV to be significantly associated with self-rated poor oral health and gum disease [71]. Study participants of the San Juan Overweight Adult Longitudinal Study (N = 740) had an oral aHPV infection prevalence of 5.7% and 20.3% of them had severe periodontitis [84]. Adults with severe periodontitis had higher odds (2.9) of oral HPV infection than those with none/mild disease [84]. Similarly, adults with clinical attachment loss >/ = 7 mm and pocket depth >/ = 6 mm had a 2- to 3-fold higher risk of having aHPV infection [84]. The presence of oral salivary HPV has been shown to be positively correlated with poor oral health [69].

### 2.4 Human immunodeficiency virus

Human Immunodeficiency virus (HIV) is the most notable acquired Immunodeficiency among men and women worldwide. Although a previously fatal disease; with the advent of anti-retroviral therapies, the outlook for People Living with HIV (PLWH) is much less bleak. Nevertheless, it is expected that those with an impaired immune system would be more susceptible to HPV infection. This hypothesis is validated in the literature. In a recent study by Riddell et al. 2022, involving 5 medical centres oral hrHPV was detected in 18% of PLWH individuals compared to 7% of HIV-negative participants. They also found that men had a higher prevalence irrespective of the HIV status (23% vs 9% in PLWH and 12% vs. 3% in HIV Negative). Notably, oral hrHPV prevalence was higher in adults with poorly controlled HIV [86]. Similarly, other studies have also documented a relatively higher prevalence of oral aHPV in PLWH 33% vs 7.14% in the HIV-negative group with no sexual risk behaviours (i.e., condomless sex) [71,78]. Notably, the presence of oral HPV was significantly linked to HIV viral load, independent of sexual risk behaviours [87].

Similar findings have been reported with regard to non-oral HPV infections in men [88].

In a cross-sectional study conducted in China by Liu et al. in 2019, investigating HPV infection of the anal canal in men, it was discovered that the prevalence was significantly influenced not only by HIV status but also by sexual orientation. In PLWH, the prevalence of HPV varied substantially with sexual orientation, with a prevalence of 30.6% in heterosexuals, 63.6% in bisexuals and 74.1% in homosexuals. This is a stark contrast to HIV-negative men, where the prevalence was 8.3% (heterosexual), 23.8% (bisexual) and 29.2% (homosexual). Of interest, further analysing the 45–71-year-old group, the prevalence of hrHPV was 35.3% in the PLWH group compared to 28.6% in the HIV-negative group [88]. Not only did this study identify a stark increase in HPV (especially hrHPV) in HIV-positive men, but it also ratified the correlation seen in homosexual men, as well as men over the age of 45 years. This trend was also seen by Muller et al. 2016 in a South African cohort of patients who were HIV positive and of the 44% who were MSM, 91.8% had an anal HPV infection with 81.2% being hrHPV. Furthermore, they also recognized that being HIV-positive constituted an independent risk factor for acquiring an anal HPV infection [89].

### 2.5 Cigarette smoking

Cigarette smoking has been shown to be positively correlated with oral HPV infection. NHANES 2009–2016 Survey using 13,089 participants reported a higher prevalence of oral hrHPV in current smokers (6.7%) compared to non-smokers (2.6%), irrespective of gender. In men, oral hrHPV was detected in 10.5% of smokers as opposed to 4.5% in non-smokers and 2.1% in women smokers as opposed to 0.9% in non-smokers [90]. Though the exact mechanism of how smoking results in higher rates of HPV infection is not yet known, it is likely due to impaired innate cellular immunological function and injury to mucosa from the toxic substances contained in cigarettes combined with those produced from combustion [91].

### 2.6 Demographics

It is clear by now that the combination of risk factors leads to oral HPV prevalence which then leads to malignant transformation. From the collation of available studies, the individual with the highest risk would be a homosexual male with multiple partners, who is over the age of 45, with HIV, and an active smoker with poor oral hygiene. Those who are at an elevated risk would be men who have one or more of those identified risk factors.

## 3 Risk factors associated with the persistence of oral HPV

In the realm of cervical cancer research, it is firmly established that persistent infection with hr-HPV stands as the primary factor contributing to the development of cervical cancer. The detection of an infection at a specific moment through an oral sample or serum is insufficient to predict the development of oropharyngeal malignancy. Instead, the realization of the virus's unintentional malignant potential requires persistent infection over time. However, there is a dearth of research on persistence, and additional studies are necessary to make a meaningful determination of the factors that predispose individuals to oral HPV persistence.

### 3.1 Human immunodeficiency virus

HIV not only leads to a higher acquisition risk of oral HPV but also leads to a reduced clearance rate and thus persistence. Riddell et al. 2022 reported that the median clearance time for oral HPV in HIV-negative individuals was 3–4 months compared to 16 months for HIV-positive individuals [86]. They also noted that CD4^+^ T-cell count <200 cells/uL, non-adherence to anti-retroviral therapy and history of acquired immune deficiency were associated with longer time taken to clear oral HPV. Importantly, those patients who were HIV positive (CD4^+^ <200 cells/uL) who had pre-existing HPV infection did not clear the virus in the 24-month study period. This was similar to the results from D'Souza et al. 2020 who followed a group of 447 men with HIV and at high risk of acquiring HIV for 7 years which showed a median clearance time of 16.8 months [81]. Furthermore, Giuliani et al. 2020 reported that those HIV-positive patients (CD4^+^ T cell <200cels/mm3 and aged over 46 years) had significantly reduced clearance of any hrHPV [82].

### 3.2 Other factors

#### 3.2.1 Sexual behaviours

Aside from HIV, there is limited available literature that looks specifically at identifying other risk factors independently associated with oral HPV persistence. The HIM (HPV in Men) trial showed short-term persistence of oral HPV in patients diagnosed with gingivitis during adulthood and inversely with the lifetime number of sexual partners. Persistence at 12 months or longer was only positively associated with the number of lifetime sexual partners [92,93]. Furthermore, Guiliani et al. 2020, found that HPV clearance declined in those with more than 6 recent oral sex partners (aHR 0.18, 95% CI 0.05 to 0.65) [82]. Similarly, a study in 2014 found circumstantial evidence that persistence increased with earlier onset of sexual activity (<13 years), early initiation of oral contraceptives and importantly oral sex [94].

#### 3.2.2 Age and gender

In the POPS (The Persistent Oral Papillomavirus Study)//MOUTH (Men and Women Understanding Throat HPV) trials by D'Souza et al., 2020, 1833 participants from PLWH and at-risk HIV-uninfected individuals in the United States, underwent 6-monthly follow-ups for 4 years using oral rinse sample collections. Participants who were persistently positive for oral HPV at the end of the 4 years were then enrolled in the POPs trial. Oral HPV-16 infections were significantly less likely to be cleared than other hrHPV types (5-year persistence 32% vs 18% HR = 1.48 95% CI 1.03 to 2.14). Infections were less likely to clear among men than women (5-year persistence 25% vs 15%, HR = 0.63, 95% CI = −0.51 to 0.79) as well as those aged 60 or over (5-year persistence >/ = 60 41% vs 11% in <40, HR = 0.42, 95% CI = 0.3 to 0.61). Furthermore, they also found men aged over 30 years had a higher incidence of hrHPV persistence at 6 months when compared to men aged 18–30 years [91].

#### 3.2.3 Cigarette smoking

Antonsson et al. (2020) discovered a link between cigarette smoking and persistent oral HPV in a general population cohort of 704 individuals aged 20 to 70 [63]. Similarly, Haukioka et al. (2014) identified this association in a subset of 60 women with persistent oral HPV infection from the Finnish Family HPV Study [95].

#### 3.2.4 Geography

In a study by Sethi et al. (2022), involving 1011 Indigenous Australians undergoing annual oral HPV testing over 24 months, the persistence of oral HPV was notably linked to living in rural areas and having a history of receiving oral sex [96].

## 4 Cervical screening program

Initially invented in the 1940s by Dr. Papanicolaou, the ‘pap smear’, revolutionized cervical cancer screening. The procedure involves collecting cervical cells with a spatula or cytobrush, smearing them on a slide, staining with Papanicolaou stain, and examining for abnormalities. This rapidly became the gold standard in cervical cancer screening [97]. In the 1990s liquid-based cytology (LBC) based methods were later established which allowed the separation of other cells that obscured the target cells [97]. Although theoretically better, LBC did not yield a higher sensitivity or specificity compared to the pap smear [98]. On the other hand, LBC-based methods streamlined the process and helped make the processing of samples more efficient. Although Pap smear-based methods are highly specific (approximately 98%), they are not sensitive (55–80%) and require repeated frequent testing [97]. Despite the forgoing limitations, this method for cervical cancer screening enabled the reduction of cervical cancer incidence and mortality by over 70% in developed countries at the turn of the century [98].

As it became known that close to 99.7% of cervical cancer was directly attributable to HPV, In 2003 the FDA approved the first HPV-DNA assay. With its implementation, in combination with a pap smear, the sensitivity rose to near 100% [5,99]. Since there is a significant lead time of 10–20 years between dysplasia and neoplasia, HPV-DNA assay was only required every 5 years in conjunction with Pap smear/LBC. Hence, the implementation of the HPV-DNA assay was a landmark in cervical cancer screening.

In fact, in 2014, the HPV-DNA assay was verified as the sole test for screening cervical cancer [100]. Subsequently, the entire surveillance paradigm shifted to be centred around the assay. It is noteworthy that despite its high sensitivity, the HPV-DNA assay has an inferior specificity at detecting cervical intraepithelial neoplasm 2+. This is secondary to the assay not being able to discriminate between transient and persistent infections at one testing interval [101]. This exposes women, particularly those under 30 with a positive HPV-DNA assay, to a likely unnecessary further procedure. For example, a study reported that 86.7% of participants with a positive HPV-DNA assay did not progress to cancer [102]. As opposed to Pap smears/LBC alone which led to overdiagnosis and treatment of low-grade squamous intra-epithelial lesions (LSIL), particularly in young women. Had these LSIL not been detected through screening they would likely have otherwise regressed without intervention. The treatment of LSIL which consists of ablative procedures has also been associated with an increased risk of long-term fertility issues [103].

In Australia, the cervical screening program now consists of 5 yearly HPV-DNA assay in those aged 25–74 years old, with the option to self-collect. This is significantly less cumbersome to the patient and healthcare system, compared to the previous 1–2 yearly testing. To address the inferior specificity, only positive results need to be investigated further with either a Pap smear or colposcopy. This process allows delineation between cancerous lesions, precancerous lesions, and mere transient HPV infections [104].

The basis for this screening program relies on the well-researched natural history of cervical dysplasia to neoplasia. Unlike the cervix, the natural progression from high-grade dysplasia to malignancy in the oropharynx is not known [105]. This makes the screening for HPV-OPC uniquely more challenging.

## 5 Strategy to prevent the development of HPV-OPC

This review aimed to provide a comprehensive overview of the current status of HPV- OPC, with the intention to advocate for a screening program. In order to achieve this aim, first we surveyed the literature on hrHPV infection, persistence, pathogenesis and risk factors associated with HPV-OPC. Identification of specific factors governing the pathogenesis of HPV-OPC and risk factors in susceptible individuals will lay the foundation for a targeted screening program for HPV-OPC.

Currently, there is no active oral HPV screening program globally. However, the success of the cervical cancer screening program, which has been instrumental in the early diagnosis of cervical cancer, sets a good precedent [106,107]. Early diagnosis enables timely intervention, significantly reducing morbidity and mortality for the patient.

The aforementioned clinical and scientific evidence clearly demonstrates the necessity of an oral screening programme for HPV-OPC. Firstly, the commonly susceptible subsites of the oropharynx such as the base of tongue and tonsils for acquiring hrHPV are difficult to access clinically [108,109]. Therefore, most patients with HPV-OPC, present initially with a neck mass, when the tumour has spread to the ipsilateral lymph nodes. The primary lesion can then be detected with flexible nasal endoscopy (FNE) and magnetic resonance imaging (MRI). Secondly, detecting occult indolent asymptomatic disease is substantially harder even for expert ENT surgeons. Thirdly, the diagnostic procedures are substantially expensive. An alternative oral screening programme that does not burden the healthcare system is an unmet, urgent medical need. Saliva liquid biopsy offers a user-friendly, non-invasive, cost-effective platform for such an endeavour. Saliva collection is painless compared to invasive methods. In fact, patients can self-collect saliva, which may enable the extension of oral screening to a home-based assay in future. As salivary fluids invariably come into contact with the tumour site, even a small indolent lesion could release detectable HPV viral particles into the liquid biopsy [110,111].

Our research group discovered the world's first occult 2 mm HPV-OPC in an asymptomatic individual through serial saliva testing for HPV-16 [61]. In our study, 665 cancer-free men and women aged 18–89 years attending an oral health centre in Australia had salivary oral rinse samples collected and analysed for HPV-16 DNA via Polymerase chain reaction (PCR). Those that screened positive, were invited to provide another sample every six months until the PCR returned negative over a 36-month period. Of the three participants that remained persistently positive for 36 months, one had a p16INK4a expression and HPV16 DNA positive T1N0M0 tonsillar SCC [61]. This was only diagnosed after the patient underwent an elective diagnostic tonsillectomy. Due to the minuscule size of the tumour, a consort of head and neck surgeons, radiation oncologists and medical oncologists determined that the patient didn't require adjuvant chemo or radiation therapy. Post-tonsillectomy saliva samples and subsequent samples tested negative for hrHPV. Similarly, Similar observations have been reported in the study conducted by D'Souza et al. 2020. By screening 1833 participants they identified 128 to have persistent hrHPV infections and one of the participants who was positive for HPV16 over 4.5 years was later diagnosed with HPV-OPC [91]. D'Souza et al. and our study substantiate the potential scientific framework for a screening program for the detection of HPV-OPC. It is noteworthy that both of these studies were conducted without targeting specific susceptible individuals with risk factors across all ages encompassing both men and women.

The critical analysis of the literature performed in the present comprehensive review identified that middle-aged men face the highest risk of acquiring an oral hrHPV infection as well as persistence over an extended period of time. Hence, middle-aged men have the highest risk of developing HPV-OPC. In addition, PLWH, poor oral hygiene and active/ex-smokers are also potential risk factors for acquiring oral hrHPV infection. It is important to obtain information on patients’ sexual behaviour, specifically pertaining to oral sex as they are at the greatest risk of developing HPV-OPC. Targeting a specific cohort of susceptible patients with risk factors enables the design of a more effective oral screening program. Such initiative will certainly lead to better patient outcomes as well as alleviate the healthcare burden associated with advanced-stage cancer treatment.

Nevertheless, implementation of a screening program is hindered by the absence of the following: firstly, there is limited data on the natural history of oral hrHPV specifically in this ‘high-risk group’; secondly, it's unclear at what frequency sampling should occur (multiple collections over a set period vs. single collections); thirdly, the sensitivity and specificity of screening tools are yet to be rigorously verified; fourthly, an appropriate method for accurate diagnosis of pre-malignant lesions in patients who remain persistently positive for oral hrHPV had yet to be determined and lastly, the lack of robust evidence that early diagnosis and treatment will truly lead to a significant reduction in morbidity and mortality [112,113]. While there are still barriers to overcome, the potential for an oral screening program for HPV-OPC to emulate the success of the cervical screening program remains a very real prospect.

With this prospect on the horizon, we are presently engaged in a large-scale trial to assess the efficacy of salivary diagnostics in preventing HPV-associated oropharyngeal cancer in men.

## CRediT authorship contribution statement

**Akila Wijesekera:** Writing – review & editing, Writing – original draft, Visualization, Validation, Resources, Project administration, Methodology, Investigation, Formal analysis, Data curation, Conceptualization. **Chameera Ekanayake Weeramange:** Writing – review & editing, Writing – original draft, Validation, Supervision, Resources, Project administration, Methodology, Investigation, Formal analysis, Data curation, Conceptualization. **Sarju Vasani:** Writing – review & editing, Writing – original draft, Validation, Supervision, Methodology, Conceptualization. **Liz Kenny:** Writing – review & editing, Validation, Supervision, Project administration, Conceptualization. **Emma Knowland:** Supervision, Formal analysis, Data curation. **Jayampath Seneviratne:** Writing – review & editing, Writing – original draft, Supervision, Project administration, Methodology, Investigation. **Chamindie Punyadeera:** Writing – review & editing, Writing – original draft, Visualization, Validation, Supervision, Resources, Project administration, Methodology, Investigation, Formal analysis, Data curation, Conceptualization.

## Declaration of competing interest

The authors declare that they have no known competing financial interests or personal relationships that could have appeared to influence the work reported in this paper.

## Data Availability

No data was used for the research described in the article.

## References

[1] Strauss M.J., Shaw E.W. (1949). Crystalline virus-like particles from skin papillomas characterized by intranuclear inclusion bodies. Proc. Soc. Exper. Biol. Med. Soc. Exper. Biol. Med. (New York, N.Y.).

[2] Crawford L.V., Crawford E.M. (1963). A comparative study of polyoma and papilloma viruses. Virology.

[3] Human papillomavirus (HPV) – immunisation. Vic.gov.au. https://www.betterhealth.vic.gov.au/health/healthyliving/human-papillomavirus-hpv-immunisation.

[4] McMurray H.R., Nguyen D., Westbrook T.F., McAnce D.J. (2001). Biology of human papillomaviruses. Int. J. Exp. Pathol..

[5] Burd E.M. (2003). Human papillomavirus and cervical cancer. Clin. Microbiol. Rev..

[6] Lang Kuhs K.A., Faden D.L., Chen L. (2022). Genetic variation within the human papillomavirus type 16 genome is associated with oropharyngeal cancer prognosis. Ann. Oncol..

[7] Chaturvedi A.K., Engels E.A., Anderson W.F., Gillison M.L. (2008). Incidence trends for human papillomavirus-related and -unrelated oral squamous cell carcinomas in the United States. J. Clin. Oncol..

[8] Rettig E.M., Fakhry C., Khararjian A., Westra W.H. (2018). Age profile of patients with oropharyngeal squamous cell carcinoma. JAMA Otolaryngol. Head Neck Surg..

[9] Garset‐Zamani M., Grønhøj C., Jensen D.H. (2020). The current epidemic of HPV-associated oropharyngeal cancer: an 18-year Danish population-based study with 2,169 patients. Eur. J. Cancer.

[10] Rettig E., Kiess A.P., Fakhry C. (2015). The role of sexual behavior in head and neck cancer: implications for prevention and therapy. Expert Rev. Anticancer Ther..

[11] Anantharaman D., Marron M., Lagiou P. (2011). Population attributable risk of tobacco and alcohol for upper aerodigestive tract cancer. Oral Oncol..

[12] Kreimer A.R., Shiels M.S., Fakhry C., Johansson M., Pawlita M., Brennan P. (2018). Screening for human papillomavirus-driven oropharyngeal cancer: considerations for feasibility and strategies for research. Cancer.

[13] Canfell PK. An Australian success story: the HPV vaccine. Cancer Council NSW. https://www.cancercouncil.com.au/news/australian-success-story-hpv-vaccine/. Published October 30, 2023.

[14] De Souza M.M.A., Härtel G., Olsen C.M., Whiteman D.C., Antonsson A. (2023). Oral human papillomavirus (HPV) infection and HPV vaccination in an Australian cohort. Int. J. Cancer.

[15] Zhang Y., Fakhry C., D'Souza G. (2021). Projected association of human papillomavirus vaccination with oropharynx cancer incidence in the US, 2020-2045. JAMA Oncol..

[16] Chesson H.W., Dunne E.F., Hariri S., Markowitz L.E. (2014). The estimated lifetime probability of acquiring human papillomavirus in the United States. Sex. Transm. Dis..

[17] Fu L., Sun Y., Han M., Wang B., Xiao F., Zhou Y., Gao Y., Fitzpatrick T., Yuan T., Li P., Zhan Y., Lu Y., Luo G., Duan J., Hong Z., Fairley C.K., Zhang T., Zhao J., Zou H. (2022). Incidence trends of five common sexually transmitted infections excluding HIV from 1990 to 2019 at the global, regional, and national levels: results from the global burden of disease study 2019. Front. Med..

[18] World Health Organization: WHO. Sexually transmitted infections (STIs). https://www.who.int/news-room/fact-sheets/detail/sexually-transmitted-infections-(stis)#:~:text=STIs%20have%20a%20profound%20impact,and%20trichomoniasis%20(156%20million). Published July 10, 2023.

[19] Burchell A., Winer R.L., De Sanjosé S., Franco E.L. (2006). Chapter 6: epidemiology and transmission dynamics of genital HPV infection. Vaccine.

[20] (2006). Genital human papillomavirus infection in men. ScienceDirect.

[21] Kjaer S.K., Chackerian B., van den Brule A.J., Svare E.I., Paull G., Walbomers J.M., Schiller J.T., Bock J.E., Sherman M.E., Lowy D.R., Meijer C.L. (2001). High-risk human papillomavirus is sexually transmitted: evidence from a follow-up study of virgins starting sexual activity (intercourse). Cancer epidemiology, biomarkers & prevention : a publication of the American Association for Cancer Research. American Society of Preventive Oncology.

[22] Doorbar J., Quint W., Banks L. (2012). The biology and life-cycle of human papillomaviruses. Vaccine.

[23] Clifford G.M., Smith J.S., Plummer M., Muñoz N., Franceschi S. (2003 Jan 13). Human papillomavirus types in invasive cervical cancer worldwide: a meta-analysis. Br. J. Cancer.

[24] Doorbar J. (2005). The papillomavirus life cycle. J. Clin. Virol..

[25] Dyson N., Howley P.M., Münger K., Harlow E. (1989). The human papilloma virus-16 E7 oncoprotein is able to bind to the retinoblastoma gene product. Science.

[26] Venuti A., Paolini F., Nasir L., Corteggio A., Roperto S., Campo M.S., Borzacchiello G. (2011). Papillomavirus E5: the smallest oncoprotein with many functions. Mol. Cancer.

[27] Estêvão D., Costa N.R., Gil da Costa R.M., Medeiros R. (2019). Hallmarks of HPV carcinogenesis: the role of E6, E7 and E5 oncoproteins in cellular malignancy. Biochimica et Biophysica Acta (BBA) - Gene Regulatory Mechanisms.

[28] Longworth M.S., Laimins L.A. (2004 Jun). Pathogenesis of human papillomaviruses in differentiating epithelia. Microbiol. Mol. Biol. Rev..

[29] Chai R.C., Lambie D., Verma M., Punyadeera C. (2015). Current trends in the etiology and diagnosis of HPV-related head and neck cancers. Cancer Med..

[30] Ekanayake Weeramange C., Tang K.D., Vasani S., Langton-Lockton J., Kenny L., Punyadeera C. (2020). DNA methylation changes in human papillomavirus-driven head and neck cancers. Cells.

[31] Fischer M., Uxa S., Stanko C., Magin T.M., Engeland K. (2017). Human papilloma virus E7 oncoprotein abrogates the p53-p21-DREAM pathway. Sci. Rep..

[32] Pal A., Kundu R. (2020). Human papillomavirus E6 and E7: the cervical cancer hallmarks and targets for therapy. Front. Microbiol..

[33] Yuan X., Larsson C., Xu D. (2019). Mechanisms underlying the activation of TERT transcription and telomerase activity in human cancer: old actors and new players. Oncogene.

[34] Moody C.A., Laimins L.A. (2010). Human papillomavirus oncoproteins: pathways to transformation. Nat. Rev. Cancer.

[35] Duensing S., Lee L.Y., Duensing A., Basile J., Piboonniyom S., Gonzalez S., Crum C.P., Munger K. (2000). The human papillomavirus type 16 E6 and E7 oncoproteins cooperate to induce mitotic defects and genomic instability by uncoupling centrosome duplication from the cell division cycle. Proc. Natl. Acad. Sci. USA.

[36] Syrjänen S.M., Syrjänen K.J. (1999). New concepts on the role of human papillomavirus in cell cycle regulation. Ann. Med..

[37] Lechner M., Liu J., Masterson L., Fenton T.R. (2022). HPV-associated oropharyngeal cancer: epidemiology, molecular biology and clinical management. Nat. Rev. Clin. Oncol..

[38] Fakhry C., Fung N., Tewari S.R., D'Souza G. (2020). Unique role of HPV16 in predicting oropharyngeal cancer risk more than other oncogenic oral HPV infections. Oral Oncol..

[39] LeConte B.A., Szaniszlo P., Fennewald S.M., Lou D.I., Qiu S., Chen N.-W., Lee J.H., Resto V.A. (2018). Differences in the viral genome between HPV-positive cervical and oropharyngeal cancer. PLoS One.

[40] Lang Kuhs K.A., Faden D.L., Chen L., Smith D.K., Pinheiro M., Wood C.B., Davis S., Yeager M., Boland J.F., Cullen M., Steinberg M., Bass S., Wang X., Liu P., Mehrad M., Tucker T., Lewis J.S., Ferris R.L., Mirabello L. (2022). Genetic variation within the human papillomavirus type 16 genome is associated with oropharyngeal cancer prognosis. Ann. Oncol.: Official Journal of the European Society for Med. Oncol..

[41] Mirabello L., Yeager M., Cullen M., Boland J.F., Chen Z., Wentzensen N., Zhang X., Yu K., Yang Q., Mitchell J., Roberson D.A., Bass S., Xiao Y., Burdett L., Raine-Bennett T., Lorey T., Castle P.E., Burk R.D., Schiffman M. (2016).

[42] Agalliu I., Gapstur S.M., Chen Z. (2016). Associations of oral α-, β-, and γ-human papillomavirus types with risk of incident head and neck cancer. JAMA Oncol..

[43] Giuliano A.R., Lee J., Fulp W.J. (2011). Incidence and clearance of genital human papillomavirus infection in men (HIM): a cohort study. Lancet.

[44] De Sanjosé S., Brotons M., Pavón M.Á. (2018). The natural history of human papillomavirus infection. Best Pract. Res. Clin. Obstet. Gynaecol..

[45] Liu Z., Nyitray A.G., Hwang L.Y. (2018). Acquisition, persistence, and clearance of human papillomavirus infection among male virgins residing in Brazil, Mexico, and the United States. J. Infect. Dis..

[46] Scott M., Nakagawa M., Moscicki A.B. (2001). Cell-mediated immune response to human papillomavirus infection. Clin. Diagn. Lab. Immunol..

[47] Munger K., Baldwin A., Edwards K.M., Hayakawa H., Nguyen C.L., Owens M., Grace M., Huh K. (2004). Mechanisms of human papillomavirus-induced oncogenesis. J. Virol..

[48] Stanley M. (2008). Immunobiology of HPV and HPV vaccines. Gynecol. Oncol..

[49] Fahey L.M., Raff A.B., Da Silva D.M., Kast W.M. (2009). A major role for the minor capsid protein of human papillomavirus type 16 in immune escape. J. Immunol..

[50] Ghittoni R., Accardi R., Hasan U., Gheit T., Sylla B., Tommasino M. (2010). The biological properties of E6 and E7 oncoproteins from human papillomaviruses. Virus Gene..

[51] O'Brien P.M., Saveria Campo M. (2002). Evasion of host immunity directed by papillomavirus-encoded proteins. Virus Res..

[52] Stanley M.A. (2009). Immune responses to human papilloma viruses. Indian J. Med. Res..

[53] Bodily J., Laimins L.A. (2011). Persistence of human papillomavirus infection: keys to malignant progression. Trends Microbiol..

[54] Münger K., Baldwin A., Edwards K.M. (2004). Mechanisms of human papillomavirus-induced oncogenesis. J. Virol..

[55] Kreimer A.R., Johansson M., Yanik E.L. (2017). Kinetics of the human papillomavirus type 16 E6 antibody response prior to oropharyngeal cancer. J. Natl. Cancer Inst..

[56] Kreimer A.R., Brennan P., Lang Kuhs K.A. (2015). Human papillomavirus antibodies and future risk of anogenital cancer: a nested case-control study in the European prospective investigation into cancer and nutrition study. J. Clin. Oncol..

[57] Kreimer A.R., Johansson M., Waterboer T. (2013). Evaluation of human papillomavirus antibodies and risk of subsequent head and neck cancer. J. Clin. Oncol..

[58] Busch C., Hoffmann A., Viarisio D. (2022). Detection of stage I HPV-driven oropharyngeal cancer in asymptomatic individuals in the Hamburg City Health Study using HPV16 E6 serology – a proof-of-concept study. EClinicalMedicine.

[59] Koka S., Beebe T.J., Merry S.P., DeJesus R.S., Berlanga L.D., Weaver A.L., Montori V.M., Wong D.T. (2008 Jun). The preferences of adult outpatients in medical or dental care settings for giving saliva, urine or blood for clinical testing. J. Am. Dent. Assoc..

[60] Agalliu I., Gapstur S.M., Chen Z. (2016). Associations of oral α-, β-, and γ-human papillomavirus types with risk of incident head and neck cancer. JAMA Oncol..

[61] Tang K.D., Vasani S., Taheri T. (2020). An occult HPV-Driven oropharyngeal squamous cell carcinoma discovered through a saliva test. Front. Oncol..

[62] Giuliano A.R., Felsher M., Waterboer T., Mirghani H., Mehanna H., Roberts C., Chen Y.-T., Lynam M., Pedrós M., Sanchez E., Sirak B., Surati S., Alemany L., Morais E., Pavón M.A. (2023). Oral human papillomavirus prevalence and genotyping among a healthy adult population in the US. JAMA Otolaryngol.–Head Neck Surg..

[63] Antonsson A., De Souza M.M.A., Wood Z. (2020). Natural history of oral HPV infection: Longitudinal analyses in prospective cohorts from Australia. Int. J. Cancer.

[64] Brouwer A.F., Campredon L.P., Walline H.M. (2022). Prevalence and determinants of oral and cervicogenital HPV infection: baseline analysis of the Michigan HPV and Oropharyngeal Cancer (MHOC) cohort study. PLoS One.

[65] Brouwer A.F., Eisenberg M.C., Carey T.E., Meza R. (2019). Multisite HPV infections in the United States (NHANES 2003–2014): an overview and synthesis. Prev. Med..

[66] Sonawane K., Suk R., Chiao E.Y. (2017). Oral human papillomavirus infection: differences in prevalence between sexes and concordance with genital human papillomavirus infection, NHANES 2011 to 2014. Ann. Intern. Med..

[67] Hernandez A.L., Karthik R., Sivasubramanian M. (2021). Prevalence of oral human papillomavirus infection among Indian HIV-positive men who have sex with men: a cross-sectional study. BMC Infect. Dis..

[68] Sonawane K., Shyu S.S., Damgacioğlu H., Li R., Nyitray A.G., Deshmukh A.A. (2022). Prevalence and concordance of oral and genital HPV by sexual orientation among US men. JNCI Cancer Spectr..

[69] Sun C.X., Bennett N., Tran P., Tang K.D., Lim Y., Frazer I., Samaranayake L., Punyadeera C. (2017 Mar 1). A pilot study into the association between oral health status and human papillomavirus-16 infection. Diagnostics.

[70] Tang K.D., Vasani S., Menezes L., Taheri T., Walsh L.J., Hughes B.G.M., Frazer I.H., Kenny L., Scheper G.C., Punyadeera C. (2020 Oct). Oral HPV16 DNA as a screening tool to detect early oropharyngeal squamous cell carcinoma. Cancer Sci..

[71] Bui T.C., Markham C., Ross M.W., Mullen P.D. (2013). Examining the association between oral health and oral HPV infection. Cancer Prev. Res..

[72] Kreimer A.R., Pierce Campbell C.M., Lin H.-Y., Fulp W., Papenfuss M.R., Abrahamsen M., Hildesheim A., Villa L.L., Salmerón J.J., Lazcano-Ponce E., Giuliano A.R. (2013). Incidence and clearance of oral human papillomavirus infection in men: the HIM cohort study. Lancet.

[73] Bajos N., Bozon M., Beltzer N. (2010). Changes in sexual behaviours: from secular trends to public health policies. AIDS.

[74] NSFG - Listing N - key statistics from the national Survey of family growth. https://www.cdc.gov/nchs/nsfg/key_statistics/n-keystat.htm..

[75] Hernandez B.Y., Wilkens L.R., Zhu X. (2008). Transmission of human papillomavirus in heterosexual couples. Emerg. Infect. Dis..

[76] Bleeker M., Hogewoning C.J.A., Berkhof J. (2005). Concordance of specific human papillomavirus types in sex partners is more prevalent than would Be expected by chance and is associated with increased viral loads. Clin. Infect. Dis..

[77] Klein S.L., Flanagan K.L. (2016). Sex differences in immune responses. Nat. Rev. Immunol..

[78] Windon M.J., Waterboer T., Hillel A.T. (2019). Sex differences in HPV immunity among adults without cancer. Hum. Vaccines Immunother..

[79] Wira C.R., Rossoll R.M. (1995). Antigen-presenting cells in the female reproductive tract: influence of sex hormones on antigen presentation in the vagina. Immunology.

[80] D'Souza G., Agrawal Y., Halpern J., Bodison S.A., Gillison M.L. (2009). Oral sexual behaviors associated with prevalent oral human papillomavirus infection. J. Infect. Dis..

[81] D'Souza G., McNeel T.S., Fakhry C. (2017). Understanding personal risk of oropharyngeal cancer: risk-groups for oncogenic oral HPV infection and oropharyngeal cancer. Ann. Oncol..

[82] Giuliani M., Rollo F., Vescio M.F. (2020). Oral human papillomavirus infection in HIV-infected and HIV-uninfected MSM: the OHMAR prospective cohort study. Sex. Transm. Infect..

[83] Hormia M., Willberg J., Ruokonen H., Syrjänen S. (2005). Marginal periodontium as a potential reservoir of human papillomavirus in oral mucosa. J. Periodontol..

[84] Ortiz A.P., González D., Vivaldi-Oliver J. (2018). Periodontitis and oral human papillomavirus infection among Hispanic adults. Papillomavirus Res..

[85] Dalla Torre D., Burtscher D., Sölder E., Rasse M., Puelacher W. (2019). The correlation between the quality of oral hygiene and oral HPV infection in adults: a prospective cross-sectional study. Clin. Oral Invest..

[86] Riddell J., Brouwer A.F., Walline H.M. (2022). Oral human papillomavirus prevalence, persistence, and risk-factors in HIV-positive and HIV-negative adults. Tumour Virus Res..

[87] Visalli G., Di Pietro A., Currò M. (2021). How much does HIV positivity affect the presence of oral HPV? A molecular epidemiology Survey. Int. J. Environ. Res. Publ. Health.

[88] Liu X., Lin H., Chen X. (2019). Prevalence and genotypes of anal human papillomavirus infection among HIV-positive vs. HIV-negative men in Taizhou, China. Epidemiol. Infect..

[89] Müller E., Rebe K., Chirwa T., Struthers H., McIntyre J., Lewis D.A. (2016). The prevalence of human papillomavirus infections and associated risk factors in men-who-have-sex-with-men in Cape Town, South Africa. BMC Infect. Dis..

[90] Jiang C., Chen Q., Xie M. (2020). Smoking increases the risk of infectious diseases: a narrative review. Tob. Induc. Dis..

[91] D'Souza G., Clemens G., Strickler H.D. (2020). Long-term persistence of oral HPV over 7 years of follow-up. JNCI Cancer Spectr..

[92] Bettampadi D., Sirak B.A., Abrahamsen M.E. (2021). Factors associated with persistence and clearance of high-risk oral human papillomavirus (HPV) among participants in the HPV infection in men (HIM) study. Clin. Infect. Dis..

[93] iuliano A.R., Lazcano-Ponce E., Villa L.L. (2008). The human papillomavirus infection in men study: human papillomavirus prevalence and type distribution among men residing in Brazil, Mexico, and the United States. Cancer Epidemiol. Biomarkers Prev..

[94] Rautava J., Willberg J., Louvanto K. (2012). Prevalence, genotype distribution and persistence of human papillomavirus in oral mucosa of women: a six-year follow-up study. PLoS One.

[95] Haukioja A., Asunta M., Söderling E., Syrjänen S. (2014). Persistent oral human papillomavirus infection is associated with smoking and elevated salivary immunoglobulin G concentration. J. Clin. Virol..

[96] Sethi S., Ju X., Antonsson A. (2022). Oral HPV infection among indigenous Australians; incidence, persistence, and clearance at 12-month follow-up. Cancer Epidemiol. Biomarkers Prev..

[97] Bedell S.L., Goldstein L.S., Goldstein A.R., Goldstein A.T. (2020). Cervical cancer screening: past, present, and future. Sexual Med. Rev..

[98] Shingleton H.M., Patrick R.L., Johnston W.W., Smith R.A. (1995). The current status of the Papanicolaou smear. CA A Cancer J. Clin..

[99] Martin-Hirsch P.L., Wood N.J. (2011). Cervical cancer. BMJ Clin. Evid..

[100] McCarthy M. (2014). FDA panel recommends DNA test as first line cervical cancer screening test. BMJ.

[101] Ronco G., Dillner J., Elfström K.M., Tunesi S., Snijders P.J.F., Arbyn M., Kitchener H., Segnan N., Gilham C., Giorgi-Rossi P., Berkhof J., Peto J., Meijer C.J.L.M. (2014). Efficacy of HPV-based screening for prevention of invasive cervical cancer: follow-up of four European randomised controlled trials. Lancet.

[102] Chen H.-C., Schiffman M., Lin C.-Y., Pan M.-H., You San Lin, Chuang L.-C., Hsieh C.-Y., Liaw K.-L., Hsing A.W., Chen C.-J. (2011).

[103] (2023). Cervical cancer screening (PDQ®) - NCI. http://Www.cancer.gov.

[104] Health (2019). How cervical screening works. Australian government department of health and aged care. https://www.health.gov.au/our-work/national-cervical-screening-program/getting-a-cervical-screening-test/how-cervical-screening-works.

[105] van der Waal I. (2009). Potentially malignant disorders of the oral and oropharyngeal mucosa; terminology, classification and present concepts of management. Oral Oncol..

[106] Landy R., Pesola F., Castañón A., Sasieni P. (2016). Impact of cervical screening on cervical cancer mortality: estimation using stage-specific results from a nested case-control study. Br. J. Cancer.

[107] Su S.-Y., Huang J.-Y., Ho C.-C., Liaw Y.-P. (2013). Evidence for cervical cancer mortality with screening program in Taiwan, 1981–2010: age-period-cohort model. BMC Publ. Health.

[108] Chaturvedi A.K., Engels E.A., Pfeiffer R.M., Hernandez B.Y., Xiao W., Kim E. (2011). Human papillomavirus and rising oropharyngeal cancer incidence in the United States. J. Clin. Oncol.: Off. J. Am. Soc. Clin. Oncol..

[109] Nasman A., Attner P., Hammarstedt L., Du J., Eriksson M., Giraud G. (2009). Incidence of human papillomavirus (HPV) positive tonsillar carcinoma in Stockholm, Sweden: an epidemic of viral-induced carcinoma?. Int. J. Cancer.

[110] Punyadeera C., Slowey P.D. (2019).

[111] Wan Y., Vagenas D., Salazar C. (2017). Salivary miRNA panel to detect HPV-positive and HPV-negative head and neck cancer patients. Oncotarget.

[112] Vasani S. Determining the utility of a screening program to reduce the incidence of HPV driven oropharyngeal cancer. Oncoscience. https://www.oncoscience.us/article/541/text/. Published August 2021. Accessed November 5, 2023.. 10.18632/oncoscience.541 PMC8351916 34386547

[113] Kreimer A.R., Shiels M.S., Fakhry C., Johansson M., Pawlita M., Brennan P. (2018). Screening for human papillomavirus-driven oropharyngeal cancer: considerations for feasibility and strategies for research. Cancer.

